# Malaria elimination transmission and costing in the Asia-Pacific: Developing an investment case

**DOI:** 10.12688/wellcomeopenres.14769.2

**Published:** 2020-01-14

**Authors:** Rima Shretta, Sheetal Prakash Silal, Olivier J. Celhay, Chris Erwin Gran Mercado, Shwe Sin Kyaw, Anton Avancena, Katie Fox, Brittany Zelman, Ranju Baral, Lisa Jane White, Richard James Maude

**Affiliations:** 1 Global Health Group, University of California, San Francisco, 550 16th St, 3rd Floor, Box 1224, San Francisco, CA, 94158, USA; 2 Swiss Tropical and Public Health Institute, Socinstrasse 57, 4002 Basel, Switzerland; 3 University of Basel, Petersplatz 1, 4001 Basel, Switzerland; 4 Modelling and Simulation Hub, Africa, Department of Statistical Sciences, University of Cape Town, Rondebosch, Cape Town, 7700, South Africa; 5 Centre for Tropical Medicine and Global Health, Nuffield Department of Medicine, University of Oxford, Oxford, UK; 6 South African DST-NRF Centre of Excellence in Epidemiological Modelling and Analysis, Stellenbosch University, Cape Town, South Africa; 7 Mahidol-Oxford Tropical Medicine Research Unit, Faculty of Tropical Medicine, Mahidol University, Bangkok, Thailand; 8 Department of Tropical Hygiene, Faculty of Tropical Medicine, Mahidol University, Bangkok, Thailand; 9 Harvard TH Chan School of Public Health, Harvard University, Boston, USA

**Keywords:** malaria, elimination, financing, costs, government, donor, resource mobilization

## Abstract

**Background: **The Asia-Pacific region has made significant progress against malaria, reducing cases and deaths by over 50% between 2010 and 2015. These gains have been facilitated in part, by strong political and financial commitment of governments and donors. However, funding gaps and persistent health system challenges threaten further progress. Achieving the regional goal of malaria elimination by 2030 will require an intensification of efforts and a plan for sustainable financing. This article presents an investment case for malaria elimination to facilitate these efforts.

**Methods: **A transmission model was developed to project rates of decline of *Plasmodium falciparum* and *Plasmodium vivax* malaria and the output was used to determine the cost of the interventions that would be needed for elimination by 2030. In total, 80 scenarios were modelled under various assumptions of resistance and intervention coverage. The mortality and morbidity averted were estimated and health benefits were monetized by calculating the averted cost to the health system, individual households, and society. The full-income approach was used to estimate the economic impact of lost productivity due to premature death and illness, and a return on investment was computed.

**Results**: The study estimated that malaria elimination in the region by 2030 could be achieved at a cost of USD 29.02 billion (range: USD 23.65-36.23 billion) between 2017 and 2030. Elimination would save over 400,000 lives and avert 123 million malaria cases, translating to almost USD 90 billion in economic benefits. Discontinuing vector control interventions and reducing treatment coverage rates to 50% will result in an additional 845 million cases, 3.5 million deaths, and excess costs of USD 7 billion. Malaria elimination provides a 6:1 return on investment.

**Conclusion:** This investment case provides compelling evidence for the benefits of continued prioritization of funding for malaria and can be used to develop an advocacy strategy.

## Abbreviations

ACT: Artemisinin-based Combination Therapy; ADB: Asian Development Bank; APLMA: Asia-Pacific Leaders Malaria Alliance; ASEAN: Association of Southeast Asian Nations; GDP: Gross domestic product; Global Fund: Global Fund to Fight AIDS, Tuberculosis and Malaria; GMS: Greater Mekong Subregion; IMF: International Monetary Fund; IP: Inpatient; IRS: Indoor residual spraying; LIC: Low-income country; LLIN: Long-lasting insecticidal net; LMIC: Lower-middle-income country; MDA: Mass drug administration; MDB: Multilateral development bank; MOH: Ministry of Health; NMCP: National malaria control program; NSP: National strategic plan; OECD: Organization for Economic Cooperation and Development; OOP: Out-of-pocket; OP: Outpatient; PAR: Population at risk; PPP: Purchasing Power Parity; POR : Prevention of reintroduction; RDT: Rapid diagnostic test; ROI: Return on investment; STC: Sustainability, transition, and co-financing; UMIC: Upper-middle-income country; USD: United States dollar; VLY: Value of additional life year; WHO: World Health Organization.

## Introduction

The Asia-Pacific region has achieved significant gains against malaria over the last decade. Malaria cases and deaths have declined by more than 50% between 2010 and 2015 in the region’s 21 malaria-endemic countries ^1^. Sri Lanka was declared malaria-free in 2016, becoming only the second country in Southeast Asia, after the Maldives, to successfully eliminate malaria. Apart from India, Indonesia, Myanmar, and Thailand, malaria-endemic countries have reported reductions in malaria incidence of more than 75% since 2000. In Bhutan, China, and Timor-Leste, cases have declined by almost 100%, with less than 200 cases being reported in 2016 ^1^.

Progress in driving down malaria may be attributed to a number of factors. Strong political and financial support from governments and donors like the Global Fund to Fight AIDS, Tuberculosis and Malaria (the Global Fund) has enabled the scale-up of effective interventions to prevent, diagnose, and treat malaria. Financing for malaria in the Asia-Pacific region increased from less than USD 100 million in 2000 to about USD 415 million in 2016. Between 2006–2010, the Asia-Pacific region attracted between 12% and 21% of global malaria funding from the Global Fund to Fight AIDS, Tuberculosis and Malaria (hereafter Global Fund) ^2^. However, there has been a steady decline in external financing for malaria in several countries, particularly those that are middle-income and experience relatively lower malaria transmission ^3^. Although domestic financing for malaria has increased in many countries in the last decade, the need for malaria control and elimination far exceeds the available resources, particularly in the context of elimination where malaria is no longer perceived as a priority disease.

Despite the progress and opportunities for elimination, malaria remains a major cause of death and illness in the region, with an estimated 228 million cases being reported in 2018 ^4^. The recent gains made are fragile and investments could be lost if malaria resurges. The case for malaria elimination has never been stronger, particularly with the growing threat of antimalarial drug resistance arising from the Greater Mekong Subregion (GMS) and the risk of it spreading to other regions. However, in order to achieve a malaria-free Asia-Pacific—a goal endorsed by leaders at the highest levels though the Asia-Pacific Leaders Malaria Alliance (APLMA) ^2^—financial resources will need to be sustained ^5^. Reduced funding or political commitment has historically been linked to 75 resurgences of malaria in 61 countries since the 1930s ^6^.

Countries and partners need better estimates of the resources required to eliminate malaria in the long term, as well as evidence on the financial and economic benefits of investing in its elimination in order to advocate for more resources. The objectives of this study were to estimate the cost to achieve malaria elimination in the Asia-Pacific region by 2030; generate an investment case for malaria by estimating the economic benefits of malaria elimination and prevention of reintroduction (POR); and identify the funding gaps and explore the potential opportunities for generating additional financial resources for achieving malaria elimination goals.

### Financing for malaria in the Asia-Pacific region

**Figure 1.**
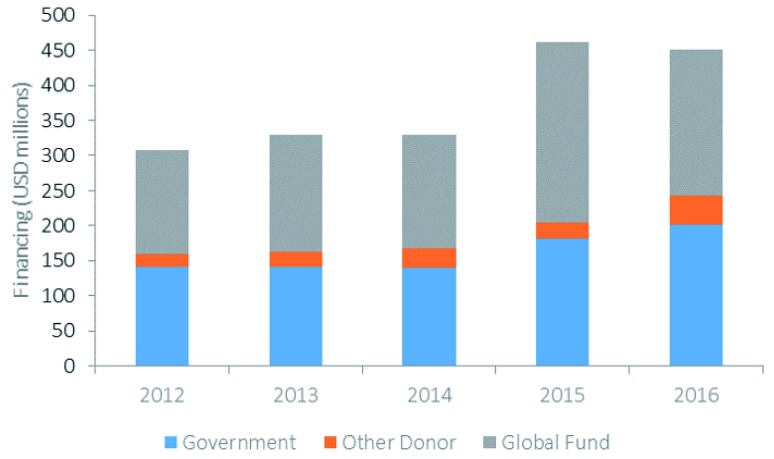
Financing for malaria in the Asia-Pacific region by source: unpublished data from the global fund.

The main sources of financing for malaria in Asia-Pacific are domestic government resources and external financing from donors. Although domestic financing for malaria has increased by over 40% in Asia-Pacific between 2015–2017 compared to 2012–2014 ^7^, most national malaria control programs (NMCPs) in the region continue to be highly reliant on external financing, particularly from the Global Fund. As Figure 1 illustrates, almost 50% of the total funding for malaria in Asia-Pacific in 2016 was from the Global Fund. This dependence on external financing is projected to continue ^8^.

## Methods

This investment case was one part of a larger study in which a transmission model ^9, 10^ and an interactive application was developed ^11^. All three papers are included in this series. The methodology specific to the investment case is included in the sections below entitled “cost projections” onwards.

The overall study design incorporated a variety of quantitative methods: numerical and regression techniques to develop a transmission model to predict the epidemiological impact of various interventions used for malaria control and elimination; and economic analysis to estimate the cost and economic impact of the interventions nationally and regionally. The population studied was the population at risk of malaria estimated by the WHO. This was computed as the proportion of the population at high and low risk of malaria (provided by the National Malaria Control Programs) applied to the United Nations population estimates to compute the number of people at risk of malaria. A combination of empirical, published data as well as expert opinion were used to develop the investment case.

### Model framework

We used outputs from a mathematical transmission model to estimate the costs and benefits of malaria elimination. The model is a dynamic compartmental model that incorporates the transmission dynamics of both *P. falciparum* and *P. vivax* malaria and interactions between the two species of malaria ^9^.

A spatially dependent version of this multi-species model was applied at a national level to the 22 countries in the Asia-Pacific. This facilitated the estimation of the relative contribution of interventions in a spatially heterogeneous transmission setting. The region was partitioned into a number of interconnected patches with each patch representing a country having its own transmission intensity. Spatial dependence between the patches was incorporated such that the risk of infection of an individual in one patch from an individual in another patch was negatively correlated with the distance between the centroids of the patches ^9, 10^.

The model was validated separately against the estimated burden of disease for *P. falciparum* and *P. vivax* (presented elsewhere ^12^) and accumulated case mortality. Several indicators (such as the estimated incidence of all malaria species and reported fatalities) were modelled for each country between 2016 and 2030, under scenario-specific assumptions. A total of 80 scenarios were simulated, based on 10 different sets of packages of interventions. These ranged from discontinuing most malaria activities to a very substantial scale-up of interventions, which could be supplemented by mass drug administration (MDA) or an increase in the coverage of long-lasting insecticide-treated nets (LLINs), at either a stable or increasing trajectory of drug resistance. While the reported coverage of interventions (particularly LLINs and indoor residual spraying (IRS)) were included in the model to inform changes in incidence, there was little available data on coverage of other interventions between 2000 and 2015, such as the introduction of community health workers). These coverage statistics were therefore imputed based on observed changes in reported incidence. The mortality predicted by the model was validated against reported deaths. A full description of the model is available elsewhere ^9, 10^.

The model estimated the impact of several intervention scenarios on the transmission of *P. falciparum* and *P. vivax* malaria from 2016 to 2030 in each of the 22 countries. Data used to calibrate and validate the model were sourced from World Malaria Reports (2001–2016) ^13– 20^ and peer reviewed literature ^21– 23^. While reported coverage of interventions (particularly LLINs and IRS) were included in the model to inform changes in incidence, there was little available data on health system advances between 2000 and 2015 (such as the introduction of community health workers); thus, these were imputed based on observed changes in reported incidence. The mortality predicted by the model was validated against reported deaths.

### Elimination scenarios

A total of 80 scenarios were generated. Four counterfactual scenarios (No. 1–4 in Table 1) including one “business as usual” scenario was modelled in which coverage remained the same as for 2016 (the last data point for which covariate rates were available for all 22 countries), and three reverse scenarios that simulated the potential impact of scaling down the malaria program. The six elimination scenarios (No. 5–10 in Table 1 were modelled sequentially to increase in complexity and in the number of interventions included.

**Table 1.** Modelled scenarios used in the transmission model.

	Scenario	Description
1	Business as usual	• Continue all interventions at 2015 levels from 2016 through 2030
2	Reverse scenario 1	• Business as usual • IRS activities ceased
3	Reverse scenario 2	• Reverse scenario 1 • Distribution of new LLINs ceased
4	Reverse scenario 3	• Reverse scenario 2 • Treatment rates reduced by 50%
5	Universal coverage	• Business as usual • Coverage of population at risk with test and treat increased from 2017 onwards in a linear fashion over eight years to 80% by 2025 • Quinine is switched to injectable artesunate for management of severe disease in 2017
6	IRS	• Universal coverage • IRS coverage in 2017 doubled in a linear fashion over eight years
7	Effective usage	• Universal coverage • Effectiveness of LLINs increased • Surveillance increased
8	New *P. vivax* treatment	• Effective usage • Replace primaquine with a new *P. vivax* treatment
9	New LLINs	• New *P. vivax* treatment • Life of LLINs doubled
10	New *P. falciparum* treatment	• New LLINs • First-line Artemisin based Combiantion Therapy (ACT) replaced with new candidate for *P. falciparum* treatment
	Assumption	Description
A	Artemisinin and ACT resistance	5% probability of treatment failure from ACTs across all countries is constant until 2018 and then increased to 30% through 2025
B	MDA	Five annual rounds of MDA at 50% coverage from 2018 starting four months before the peak of the transmission season targeted at both species
C	LLINs	Scaling up LLINs to 80% effective coverage deployed in a 3-year cycle (50%, 25% and 25%)

For each country, the minimum package of interventions that would achieve malaria elimination was determined, defined here as one year with less than one reported clinical case. This was taken to be the minimum elimination scenario for that particular country. Since the model did not distinguish between indigenous and imported cases, we assumed that certain thresholds of cases are imported, which we subtracted from the model outputs. Using Sri Lanka’s example of achieving elimination status in 2016, but reaching zero indigenous cases in October 2012, the elimination threshold was defined as the incidence per 1000 population at risk that Sri Lanka reported to WHO in 2013. This was taken to be a proxy for the level of imported cases one would expect to see in a country that has reached zero indigenous cases for the first time. This threshold was applied to the population at risk for all 22 countries.

In addition, we simulated the effect of improved targeting of malaria interventions on both costs and epidemiological outputs. We did this by reducing intervention coverage by 30% among the population at risk (PAR) for all scenarios, with and without the resistance and MDA assumptions. The outputs of averted mortality and morbidity under the elimination scenarios were expressed as reported cases and deaths (projected from reported cases) and estimated cases and deaths projected from a range of estimates. Averted cases and deaths were then used to estimate the cost, benefits, and returns on investment (ROIs).

### Additional assumptions

We applied additional assumptions to simulate various possible outcomes across all 10 scenarios: (i) The first was around the occurrence of artemisinin and ACT partner drug resistance; across all scenarios, a baseline treatment failure rate of 5% was applied across all countries from 2016–2030. Under the resistance assumption, the probability of treatment failure was kept constant at 5% through 2018 and increased to 30% between 2018 and 2025. (ii) The second assumption concerned the use of MDA. MDA was simulated as five annual rounds of dihydroartemisinin-piperaquine at 50% coverage of the population at risk from 2018 onwards, starting four months before the peak of the malaria transmission season. (iii) In a third set of simulations, LLIN scale-up was added to all the elimination scenarios in accordance with WHO guidelines for vector control, if malaria elimination was not predicted by 2030. LLIN scale-up was defined as LLIN coverage of up to 80% coverage achieved through three-year distribution cycles from 2017 to 2026. These additional rates of decline were projected separately.

These additional scenarios produced a total of 80 scenarios: with and without resistance; with and without MDA; and with and without LLIN scale up to 80%.

### PAR

Population at risk is traditionally difficult to estimate and depends on the national malaria program’s assessment of active, residual and non-active foci, among other things. The study relied on the PAR estimates reported in the World Malaria Report, as a single source for all 22 countries, though the definitions are not standardized. For all the scenarios, a declining PAR was assumed in the model. PAR values used to estimate costs in the model were adjusted to reflect the decreases in incidence predicted from the implementation of elimination-focused interventions. Historical incidence and PAR data were analysed statistically to infer a predicted change in PAR for a given change in incidence. This relationship was applied to the 2015 PAR data and updated every year until 2030 as interventions were applied in the modelled scenarios. This method has limitations, including a non-standardized definition of PAR between countries.

### Cost projections

We used the outputs of the transmission model to estimate the total costs associated with implementing each of the scenarios above. Program costs included the costs of testing and treating uncomplicated or outpatient (OP) and severe or inpatient (IP) malaria cases; vector control (i.e., LLIN distribution and IRS); supply chains; surveillance through community health workers; information, education, communication; training; MDA; new treatments (e.g., tafenoquine for *P. vivax*); and rollout of new LLINs. Unit costs for each activity were obtained using a combination of empirical data collected in various Asia-Pacific countries by the authors, literature reviews, and proxies when the previous options were unavailable (Table E1, available as extended data ^24^). From the range of costs generated, we determined the minimum, maximum, median, mean, and other percentiles of the economic benefits.

In addition, we simulated the effect of improved targeting of malaria interventions on both costs and epidemiological outputs on cost. We did this by assuming a focal deployment of interventions where the target population comprises the high risk and only 70% of the low risk population.

The total cost of the elimination scenarios was used to build this investment case. We calculated the costs to reach elimination separately for each country and then summed them to obtain the total cost for elimination in the Asia-Pacific region. To calculate the incremental or additional costs of malaria elimination (which were used to calculate ROIs), we subtracted the estimated costs of the business as usual and reverse scenarios from the elimination scenario. All monetary figures are expressed in 2015 constant USD.

### Economic benefits estimation

Using outputs from the model, we estimated the mortality and morbidity averted from malaria elimination by subtracting the estimated cases and deaths of the elimination scenario from the corresponding outputs of the “business as usual” and “reverse” scenarios. We then monetized these health benefits by looking at the averted cost to the health system, averted cost to individual households, and averted cost to society:

- Cost averted to the health system includes costs associated with diagnosis and treatment costs of IPs and OPs;
- Cost averted to the individual households is out-of-pocket (OOP) expenditures for seeking care; and
- Cost averted to the society due to patients’ lost productivity due to premature death and morbidity and caregivers’ reduced economic output.

The same cost inputs used in the cost estimation were used for calculating the economic benefits. Unit costs for case management included costs for OP visits, diagnostic tests, and drug treatments for OP malaria cases, as well as hospital hotel costs and drug treatments for IP malaria cases. OOP expenditures were estimated by applying country-specific OOP expenditure per capita separately for OP and IP cases. We calculated productivity losses among patients and caretakers by multiplying an estimate of daily productivity by the number of days lost due to illness or care seeking.

We used the full-income approach to estimate the economic impact of lost productivity due to premature death from malaria. We multiplied the number of averted deaths for each country by the country-specific values of additional life years (VLYs) and life expectancies at age 40 among males and females, which was the assumed average age of death due to malaria ^25^. One VLY was estimated to be 2.2 times the gross domestic product (GDP) per capita for each of the countries in South East Asia and the Pacific and 2.8 times the GDP per capita for each of the countries in South Asia, as suggested by the *Lancet Commission on Investing in Health* ^26^.

All costs and economic benefits were discounted at 3%.

### Return on investment

The ROI was calculated by subtracting the incremental cost of elimination from the economic benefits and dividing the resulting figure by the incremental cost of elimination. The ROI is interpreted as the economic return from every additional dollar spent on malaria elimination.

We performed the ROI analysis for 2016–2030 by comparing the elimination scenario with the business as usual and reverse scenarios under the stable and increasing resistance assumptions.

### Uncertainty analysis

We performed stochastic sensitivity analysis on the epidemiological and cost outputs of the malaria transmission model. The uncertainty interval of the epidemiological outputs was largely due to estimates of reporting coverage detailed in Maude *et al*. (2019) ^12^.

The minimum, median, and maximum malaria cases and deaths predicted by the model for each scenario were used to calculate the minimum, median, and maximum economic benefits. For the costs, we assigned an uncertainty interval of ±25% on the value of the input costs used. A total of 300 random samples were drawn, which generated a range of costs. From the range of costs generated, we determined the minimum, maximum, median, mean, and other percentiles of the economic benefits.

### Gap analysis and opportunities for resource mobilization

Using available malaria financing data in the region (donor and domestic), between 2017 and 2020, we estimated the potential gap in financing assuming the total funding envelope would remain as projected. We also assessed potential opportunities for resource mobilization to fill financing gaps by mapping private sector investors and analysing the domestic funding landscape.

## Results

### Projected declines in transmission

The transmission model predicted that malaria elimination can be achieved by all the countries in the Asia-Pacific region by 2030 by implementing a variety of scenarios. Table 2 illustrates the predicted output of the transmission model under an assumption of increasing artemisinin resistance and identifies the minimum elimination scenario defined as the scenario under which the country can achieve elimination on or before 2030 with the least amount of effort.

**Table 2.** Scenarios and predicted elimination dates.

Country	Minimum elimination scenario and interventions	MDA	LLIN	Elimination date (predicted range)	National elimination goal
Afghanistan	Effective usage	Yes	Yes	2025 (2025,2027)	None
Bangladesh	Effective usage	No	No	2025 (2024,2029)	2035
Bhutan	Effective usage	No	No	2024 (2023, 2025)	2018
Cambodia	New LLINs	Yes	No	2023 (2022, 2030)	2025
China	Business as usual (already eliminated by 2017)	No	No	2017	2020
DPRK	New *P. vivax* drug	No	Yes	2028 (2027, 2030)	2025
India	New LLINs	No	Yes	2028 (2026, 2030)	2030
Indonesia	Effective usage	Yes	No	2025 (2022,2028)	None
Lao PDR	New *P. falciparum* drug	Yes	Yes	2025 (2022,>2030)	
Malaysia	IRS	No	No No	2023 (2019, 2029)	2020
Myanmar	New *P. falciparum* drug	Yes	Yes	2025 (2024,>2030)	None
Nepal	Effective usage	No	No	2022 (2017, 2026)	2026
Pakistan	Effective usage	Yes	Yes	2022 (2021, 2030)	None
PNG	Effective usage	Yes	No	2025 (2025,2028)	
Philippines	Effective usage	No	No	2021 (2017,2023)	2030
ROK	Business as usual	No	No	2017 (2017,2019)	2017
Solomon Islands	New LLINs	Yes	No	2028(2026, 2029)	
Sri Lanka	Business as usual (already eliminated by 2017)	No	No	Already eliminated in 2013	2012
Thailand	New *P. vivax* drug	No	No	2026 (2025, 2029)	2024
Timor-Leste	Universal coverage	No	No	2019 (2017,2024)	
Vanuatu	Effective usage	Yes	No	2021 (2021, 2024)	2025
Viet Nam	Effective usage	No	No	2024 (2022, 2027)	2030

The model predicted that it is possible for all 22 countries to achieve elimination of *P. falciparum* and *P. vivax* by 2030. China, ROK, and Sri Lanka ^3^ are the only countries predicted to achieve elimination without scaling up current interventions. Elimination was predicted to be possible in Cambodia, DPRK, India, Lao PDR, Myanmar, Solomon Islands, and Thailand by 2030 using new tools and technological innovation. Elimination was predicted to be possible by 2030 through the addition of MDA in Afghanistan, Cambodia, Indonesia, Lao, Myanmar, Pakistan, PNG, Solomon Islands, and Vanuatu. In all other countries, elimination is possible with the scale up of existing interventions.

**Figure 2.**
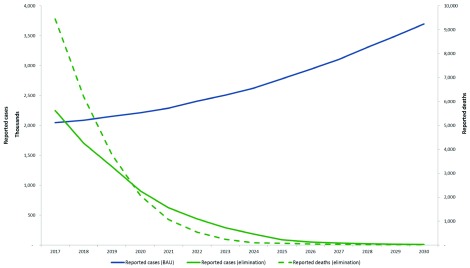
Median predicted reported cases and deaths in the Asia-Pacific region, 2017–2030 under the business as usual scenario (BAU) and elimination scenario. These are predictions projected from the *reported* cases in 2015.

Figure 2 illustrates the median reported cases and deaths between 2016–30 under the “business as usual” scenario and minimum elimination scenarios for the region. These are predictions projected from the *reported* cases in 2015.

**Figure 3.**
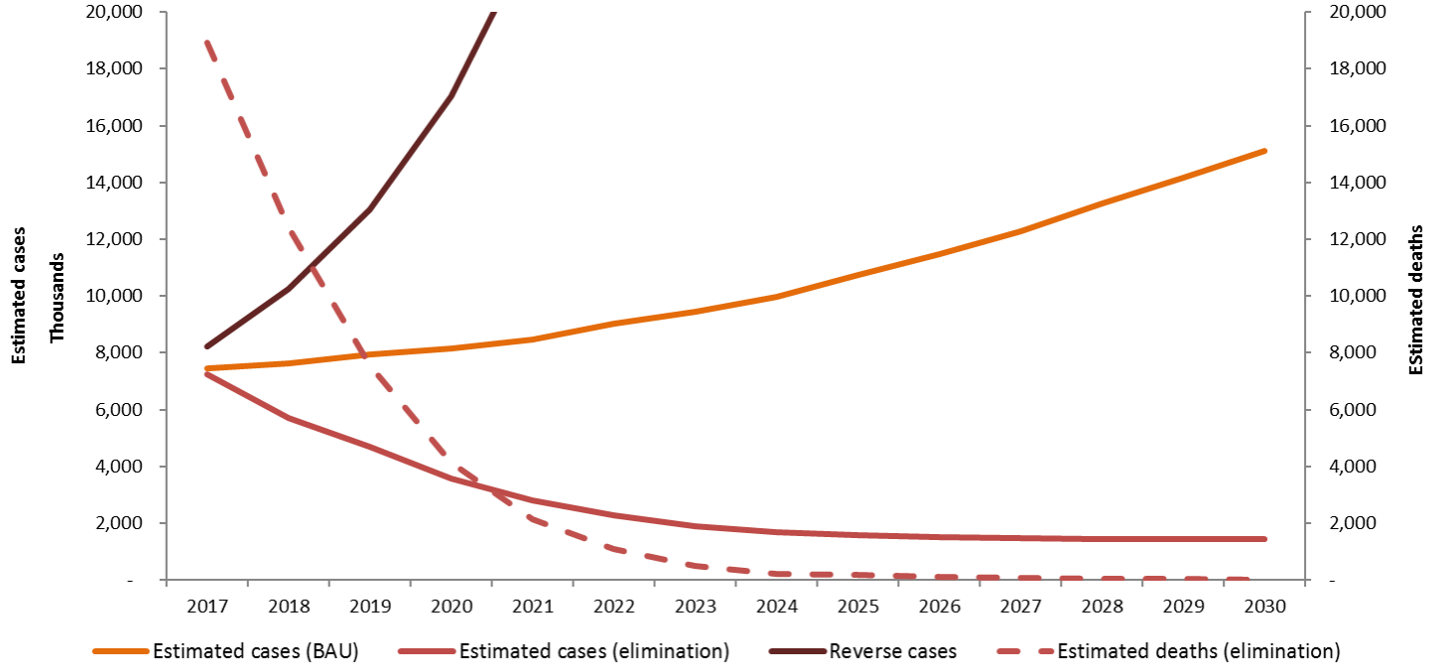
Median predicted estimated cases and deaths in the Asia-Pacific region, 2017–2030 under the “business as usual” scenario. minimum elimination and reverse scenarios.

Figure 3 Illustrates the median estimated cases and deaths between 2016 and 2030 under the “business as usual” scenario and minimum elimination scenarios for the region. These are predictions projected from the estimated cases in 2015.

In the business as usual scenario for all countries in the region, clinical cases rose from an estimated 7 million in 2016 to 15 million in 2030. Implementing the elimination scenario in each country will avert a total of over 123 million clinical cases and approximately 3.5 million deaths in the region over 14 years. In a “reverse” or worst case scenario, where interventions are halted and reduced (reverse scenario), cases increase to about 180 million by 2030. There would be about 1 billion additional cases and 3.5 million additional deaths, costing an excess of USD 7 billion between 2016–2030.

### Cost of malaria elimination through 2030

**Figure 4.**
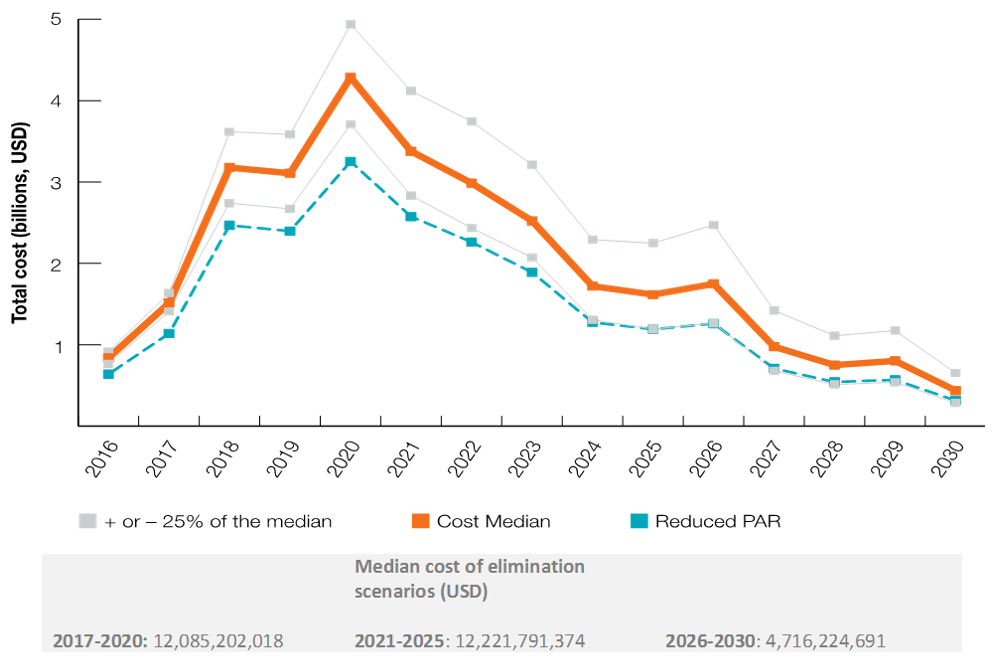
Modeled costs of the elimination scenario, 2017–2030. Median and median +/- 25% costs of the modelled minimum elimination scenarios as well as an estimate of the median cost under a scenario of reduced PAR between 2017–2030.

The cost of malaria elimination is shown in Figure 4 and Table 3 The total cost to achieve malaria elimination in the Asia-Pacific between 2017 and 2030 was estimated to be USD 29.024 billion (range: USD 23.65–36.23 billion). The median cost in 2017 for the elimination scenarios was USD 1.51 billion (range: USD 1.41–1.64 billion). Costs peak in 2020 at USD 4.29 billion (range: USD 3.71–4.94 billion), then decrease to less than USD 1 billion in 2027 (range: USD 0.68–1.42 billion) and less than USD 450 million (range: USD 0.29–0.65 billion) in 2030 when elimination is expected to be achieved in all 22 countries. Lower costs incurred are expected to continue after the elimination date as POR of malaria interventions continue. The reverse scenario would cost an excess of USD 7 billion between 2017 and 2030. If interventions were only applied to 70% of the PAR in the low-transmission areas (a crude proxy for the effect of improved targeting of interventions), the total cost would be about USD 22.49 billion.

**Table 3.** Summary of costs and benefits, 2017–2030.

Scenarios compared	Total cost (USD)	Estimated clinical cases averted	Deaths averted	Economic benefits (USD)	Incremental cost (USD)	ROI
Business as usual vs. elimination (with resistance assumption)	29.024 billion (range: 23.64–36.23)	123.14 million (estimated) ^4^ 16.54 million (reported) ^5^	386,167 (estimated) 193,084 (reported)	87.73 billion (range: 26.3–347.14)	14.05 billion	6:1
Business as usual vs. elimination (baseline)	28.953 billion (range: 23.38–35.72)	92.23 million (estimated clinical) 11.68 million (reported)	264,322 (estimated clinical) 132,161 (reported)	72.90 billion	13.79 billion	5:1
Reverse vs. elimination (with resistance assumption)	NA	845.73 million	3.487 million	N/A	6.693 billion	N/A

**Figure 5.**
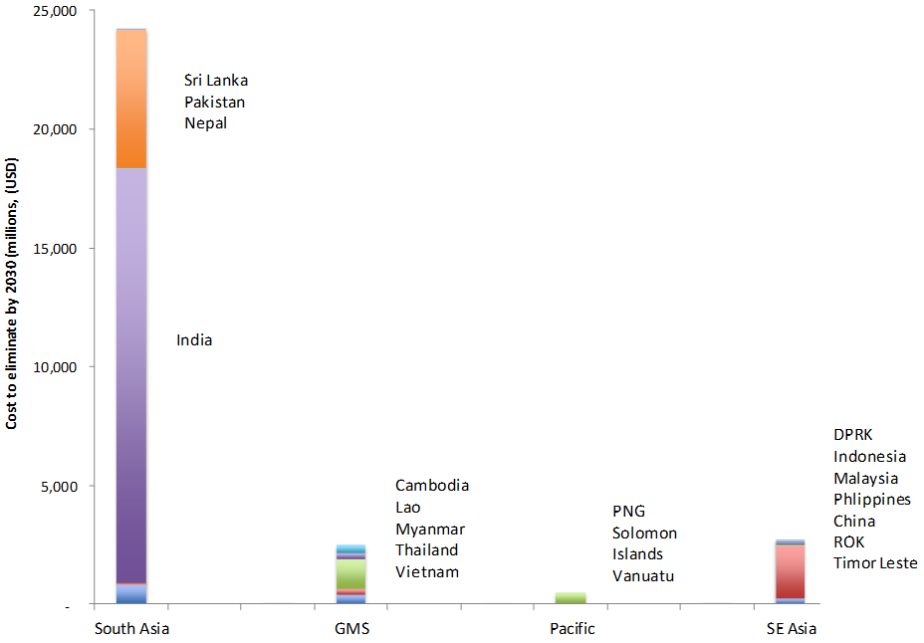
Modeled costs by country and region of the elimination scenario from 2016 to 2030.

Figure 5 illustrates how the relative costs are skewed by sub region with over 80% of the costs predicted to be incurred in South Asia, most notably, India.

### Economic benefits estimation

Compared to a business as usual scenario, interrupting local transmission can save over 400,000 lives and avert 123 million malaria cases, translating to almost USD 90 billion (range: USD 26.3–347.14 billion) in economic benefits. The economic benefits included costs averted for diagnosis and treatment costs as inpatients and outpatients, costs averted to individual and households and the monetized value of lost productivity due to premature death and morbidity and caretaker’s reduced economic output as a result of taking care of patients.

Discontinuing vector control interventions and reducing treatment coverage rates to 50% will reverse the gains made, resulting in an additional 845 million cases, 3.5 million deaths, and excess costs of USD 7 billion.

### Return on investment

The cost of malaria elimination was weighed against the epidemiological and economic costs of inaction. When the net benefits of elimination compared to the cases and costs averted in the business as usual scenario of the transmission model for the period of 2017 to 2030, the median ROI for each additional dollar invested in malaria elimination was calculated to be over 6:1. This increases to 7:1 if interventions are better targeted in low-risk areas.

### Financial gap

A median resource envelope of about USD 3 billion is needed annually to achieve elimination between 2018–2020. Total financing for the region is projected to be USD 0.5 billion annually for 2018–2020. Therefore, the anticipated gap is likely to be over 80% of the resources required for elimination between 2018–2020.

### Sensitivity analysis

**Figure 6.**
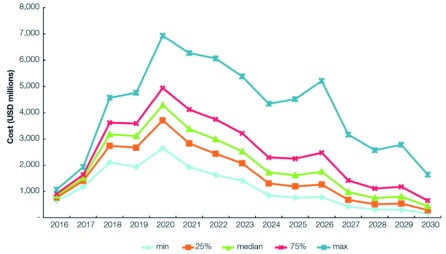
Sensitivity of the total cost to the individual cost inputs (2016–2030).

Figure 6 illustrates the sensitivity of the total cost to the individual cost inputs. At the peak in 2020, costs vary from USD 2.5 billion to USD 7 billion.

**Figure 7.**
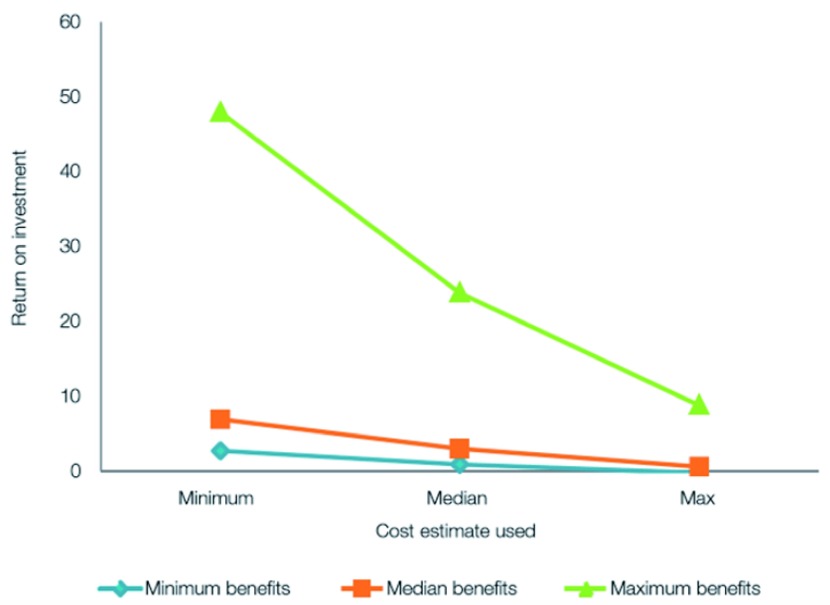
Sensitivity of the ROI to varied costs and benefits.

Figure 7 illustrates that using minimum values of the benefits will still produce a positive ROI.

## Discussion

This analysis compared the monetized value of expected benefits from malaria elimination to the investment costs over a 14-year investment period (2017–2030) in the 22 countries of the Asia-Pacific, demonstrating a robust median return of about six times the incremental investment.

The study found that by employing a variety of existing and new interventions, all countries in the Asia-Pacific could eliminate malaria by 2028—two years before the 2030 APLMA regional goal. The health, social, and economic returns are potentially formidable. Malaria elimination will save over 400,000 lives and avert over 123 million cases, translating to economic benefits of almost USD 90 billion.

Our models estimate that the total cost of achieving elimination and POR is about USD 29.02 billion (range: USD 23.64-36.23 billion) over 14 years or USD 12 billion between 2017–2020. Costs are highest in the first 4 years of efforts peaking at over USD 4 billion in 2020. These costs subsequently decline as more and more countries begin eliminating the disease and moving into POR interventions. By 2030, the cost of POR was estimated to be less than USD 500 million. Successfully achieving elimination, will however require sustained financial resources.

Using co-financing data from Global Fund concept notes, total financing for malaria was projected at USD 1.4 billion between 2018–2020, leaving an annual gap of about USD 2.5 billion or 80% of the estimated cost of elimination.

Numerous countries in the region continue to rely on Global Fund resources to provide up to 50% of their total financing for malaria elimination. However, the allocation methodology adopted by the Global Fund in 2012, utilizes a combination of disease burden and gross national income (GNI) per capita to determine the financing that countries will receive ^24^. By definition, malaria-eliminating countries have lower disease burdens, have higher incomes and are therefore a lessor priority for donors. Country-specific funding from the Global Fund to the sub-set of countries attempting to eliminate malaria has declined by over 30% ^2^. Further declines in allocations have been noted under a subsequently revised model adopted in November 2016 ^27^. Given the downward trend in malaria burden and the region’s rising economic status, this level of support is likely to be even more diminished in subsequent years.

Many malaria-eliminating countries are middle-income countries (MICs) as defined by the World Bank ^28^. The International Monetary Fund (IMF) projects average annual GDP growth rates of 3–10%, which means that economies in Asia will double or triple in size in the next decade. By 2020, four countries in Asia that are currently lower-middle-income countries (LMICs); Bhutan, Indonesia, the Philippines, Sri Lanka, will surpass the World Bank threshold for MICs of USD 4,125 GDP per capita ^24^. This means that while there is increased potential for domestic financing, more countries will also start to graduate out of aid eligibility. Of the 22 countries in the Asia-Pacific region, three are currently LICs, 15 are LMICs, and three are UMICs and one is an UIC. There are 18 currently eligible for Global Fund financing ^29^, out of which an additional two countries will be receiving the final transitional grants in the next two years (the Philippines and Sri Lanka). Political and policy changes in other donor constituencies also pose similar risks.

These changing polices have major implications for the financing and delivery of health services, for malaria elimination. Malaria financing will therefore need to depend on larger contributions from government budgets. Indeed, the expectation of the economic and health financing transition suggests that as countries develop they will spend more on health than they did before. Although domestic financing for malaria has increased by over 40% in the Asia-Pacific between 2015 and 2017 compared to 2012–2014 ^8^, the resources required far exceed the amounts available.

The potential consequences of funding gaps at this critical juncture can be serious. This analysis estimates that scaling back interventions in the Asia-Pacific could lead to an additional 3.5 million deaths, almost 1 billion cases, and economic costs of almost USD 7 billion. Emerging artemisinin resistance further threatens the gains made against malaria and regional health security with estimates of 9,560 excess deaths and USD 51 million in productivity losses annually ^30^.

To ensure uninterrupted availability of key malaria interventions, mechanisms to augment and prioritize domestic funding and improve efficiencies in the existing malaria envelope will need to be explored. The Addis Ababa Action Agenda calls on a number of resource mobilization efforts encompassing aid, domestic public resources, and support from the private sector ^31^.

Many national governments are considering raising health budgets by improving the capacity to raise tax revenue including the implementation of Pigovian or sin taxes. In the Philippines, increased taxes on tobacco and alcohol generated USD 2.3 billion within just 2 years, increasing the Department of Health budget by 63% in 2015 ^32^. This revenue has freed up resources, which would have otherwise been used for social protection of the poor. Indonesia and Vietnam have similarly implemented such revenue generating structures.

The diversification of Asia-Pacific countries’ economies presents a unique opportunity to engage the private sector in malaria elimination ^33^. Private Asian companies such as AirAsia, Samsung, the Tata group, and Alibaba have become internationally recognizable brands. Government incentives for the private sector engagement could include tax relief or tax credit schemes and policies that promote expansion or diversification of programs. For example, the Cambodian Ministry of Health has developed a policy framework for public-private partnerships in the health sector. Similarly, an airline levy such as the UNITAID model could raise more than USD 300 million per year ^34^.

The private sector may also be leveraged to provide in-kind contributions and applying their business expertise to the malaria elimination challenge. For example, in 2010, the Coca-Cola Company launched a pilot project alongside the Global Fund and the Bill & Melinda Gates Foundation to transfer core expertise to Tanzania’s Medical Stores Department, which distributes medical supplies across the country. This initiative “Project Last Mile” partnership was expanded in 2014 to include new partners with a goal to support 10 countries by 2020 ^35^.

Innovative financing options can also fill the gap between needs and resources until government budgets catch up with the financing transition. These may include health bonds, debt swaps, and blended financing mechanisms. Social impact bonds and development impact bonds are other types of instruments that have been implemented in selected settings ^36^. One example is the Mozambique Malaria Performance Bond, which is being used to raise funding from investors interested in both financial and social returns ^37, 38^. Such innovative instruments have been used to raise financing for health and other sectors, such as education and environment.

Multilateral Development Banks (MDBs) and partners can provide new financing opportunities to governments and the private sector, including cross-sectoral financing for health programs, incentivizing companies to invest in health interventions ^39^. Countries can seek out additional grants and soft-loans from MDBs to help frontload the costs of elimination. Several MDBs are currently engaged in innovative models including ADB, the Inter-American Development Bank, the Islamic Development Bank, and others in collaboration with the Bill & Melinda Gates Foundation, the Global Fund and other partners ^40– 42^.

In addition to increasing available health revenue and allocating additional resources, improved efficiencies can generate cost-savings, freeing up resources to cover financing gaps. Assessing and identifying current inefficacies and drivers of inefficiency can increase utilization of current funds.

Mathematical modelling suggests that the optimal mix of interventions will vary depending on the national or subnational setting. Reviewing efficiency of the malaria program on an annual basis, including an efficiency assessment as a pre-requisite for donor funding and linking disbursements to efficiency indictors will mitigate future inefficiencies. In-country mathematical and economic modelling could support this process and thus efforts are being made to build national modelling capacity.

In addition to pursuing additional domestic financing and meeting current co-financing requirements of existing grants, countries should appropriately plan the transition from donor to domestic funding sources 3–5 years in advance of the actual transition. Several guidance documents and tools are available to support this process ^43^.

A number of unknown factors and limitations impact the findings of this report. The costs of medicines and other interventions have been estimated based on available data and proxies were used when data were unavailable. The cost of new interventions, such as new LLINs, and new treatments such as tafenoquine, were based on historical estimates of the cost of new tools when they were first adopted rather than actual costs. In particular, separating out the cost of interventions in integrated systems is challenging and the analysts have relied on country-level partners to apportion the amounts spent on each intervention to arrive at disaggregated costs.

The cost estimates produced are highly dependent on the output of the transmission model, which was designed with a single homogeneous patch for the whole of each country, using national level data on incidence and intervention coverage. Treating the whole country as a single unit in this way is likely to lead to over-estimates in costs of elimination. Furthermore, spatial heterogeneity within each country was not modelled. These estimates are therefore subject to error, particularly in countries with heterogeneous transmission patterns. Elimination often requires targeted interventions to risk areas or populations, rather than ubiquitous coverage to an entire country. Without subnational estimates of incidence and coverage, targeted interventions are difficult to estimate and cost.

Due to the lack of suitable data, population movement was included in a rudimentary way. The transmission rate of malaria in each country was influenced by the malaria prevalence in other countries with the level of influence between countries reflecting a simple gravity model assumption. This assumption is likely to have reduced the predicted costs.

The BAU or baseline scenario refers to the range of interventions that were being implemented in 2015-the last available data point at the time of analysis. Substantial investments in the disease were made after this time particularly in the GMS through the Regional Artemisinin Initiative (RAI) grant from the Global Fund, however due to the lack of country level data on intervention coverage and incidence any progress made against the disease beyond 2015 was not built into the model. It is likely that the baseline estimates of disease incidence and cost are overestimated. Nevertheless, given the threat of drug resistance already detected in the Asia Pacific, it is not surprising that without increased efforts to combat drug resistance the BAU will no longer favour a downward trend. We were unable to predict the impact that economic development and housing improvements may have on malaria transmission or how the costs of commodities or interventions may change at the global or national levels. While we modelled for a declining PAR based on historical changes in PAR compared to changes in incidence, this method has limitations including a non-standardized definition of PAR.

While we have tried to estimate the effect that drug and insecticide resistance would have on cost, it is impossible at this stage to predict accurately the future extent and effect of drug and insecticide resistance and the actual interventions that would be implemented to address these. In addition, the impact and cost of known tools in the innovation pipeline have been modelled, however, the impact of new tools and approaches not yet developed is unknown and will be likely to decrease costs in the long term given that the cost of new tools is greatest at the time of adoption with economies of scale and competition driving costs down over time. It is also difficult to predict how the costs of interventions may change at the regional or national levels over time.

Lastly, current assessments of reported malaria incidence have limitations. Research suggests that there may be significant under-reporting in the scale of global malaria incidence and mortality due to the weakness of health reporting and information management systems as well as widespread and undocumented use of the private sector in many endemic countries. For example, the Institute for Health Metrics and Evaluation estimated a figure of 1.2 million malaria deaths in 2010—almost double the WHO’s figure of 655,000 ^44^. Similarly, a widely quoted study in *The Lancet* estimated that in India, 205,000 deaths per year could be attributed directly to malaria, which differed by more than ten times the numbers reported by the malaria program in the same year ^45^.

There have been various attempts at quantifying the true burden of malaria and more recent publications of the World Malaria Reports contain data on reported cases to health facilities as well as estimated cases based on a number of assumptions. This report utilized reported cases from the World Malaria Reports as well as estimated cases for the Asia-Pacific countries derived by Mahidol-Oxford Tropical Medicine Research Unit in collaboration with a number of partners including the WHO ^12^. These estimates were obtained by combining and triangulating data from a variety of data sources. Both reported and estimated cases are depicted in the graphs. Nevertheless, the wide variation in estimates of burden makes it harder to be sure of the resources required to eliminate the disease. Without an informed and complete understanding of the current cartography of malaria risk and prevalence, future projections of the cost of eliminating malaria face an overwhelming uncertainty.

The cost of malaria elimination in the Asia Pacific has been previously estimated by APLMA at USD 1 billion per year in the first five years of the implementation of its roadmap and just under USD 2 billion per year in subsequent phases, amounting to a total of USD 24.5 billion over 15 years. However, these costs were based mainly on transmission models whose exclusive focus was on P. falciparum malaria applying malaria transmission dynamics from Sub-Saharan African countries to predict 90% reductions in current levels of malaria-related mortality and morbidity and not malaria elimination ^7^. The impact of malaria interventions such as LLINs and IRS on *P. vivax* and other species differ vastly from what has been observed for *P. falciparum* making these previous estimates unreliable.

We believe that the benefits of elimination estimated in this paper are conservative. Beyond the benefits of achieving malaria elimination as explained in this report, other benefits are likely, but are harder to quantify as there are no reliable quantitative estimates on how malaria may impact these. As a by-product of national elimination, other positive externalities are increased tourism, a strengthened health system, better cognitive development, and improved regional health security. In addition, elimination may bring significant benefits to other regional public goods including opportunities to create stronger cross-border disease coordination.

Because of these uncertainties, estimated costs can only provide an indicative guide or baseline to help determine financing needs. It is therefore important that economic estimates are constantly reviewed in the light of new information, through to 2030. Importantly, due to the diversity of the region, further analysis is required to adapt the model to individual country settings and develop country-level estimates based on the national context. This, however, makes it even more important that funds can be put in place quickly to match currently expected costs.

Despite limitations above, this investment case provides robust evidence of the benefits of continued prioritization of funding for malaria. The ROIs remain robust, comparable to those obtained for other high impact investments such as immunization programs and cardiovascular disease research ^46^. Although the short-term investment needed may seem substantial, these must be considered in the context of other major public health, developmental projects and other national level expenditures. For example, the new High-Speed II railway system in the United Kingdom is expected to cost more than USD 70 billion, more than twice the amount for eliminating malaria from the Asia-Pacific ^47^. Similarly, in 2015, US military spending amounted to almost USD 600 billion ^48^ and spending on costumes, candy and decorations on Halloween is expected to amount to over USD 8 billion this year ^49^. On the contrary, the savings from malaria elimination will allow the treatment of 11 million outpatients for 10 years or allow the construction of over 100,000 km of roads in Asia.

Focused advocacy at all levels is needed to reach key decision-makers in order to highlight the social and economic benefits of investing in malaria elimination and the risks of not doing so. In particular, emphasis on the threat of drug resistance in undermining success, increasing costs and posing a risk of regional health security is needed. Continued engagement is needed with governments to focus attention on increased domestic budgets to reach the regional goal of a malaria-free Asia-Pacific by 2030.

## Conclusion

Global progress against malaria has been dramatic over the past decade. These gains, however, have been driven by substantial political and financial commitments by governments and external partners. Accelerating the trajectory of malaria elimination and preventing a resurgence of malaria will entail sustained commitments by all stakeholders. This requires the need for accurate estimates of the investment required in the short, medium and long term as well as a robust evidence to justify the gains from such an investment.This analysis demonstrates that the estimated cost for achieving malaria elimination is outweighed by the significant health, economic and social benefits that will ensue providing an economic return of more than six times the investment. Eliminating the disease should therefore receive a special focus for financing. Malaria is a major ongoing cost driver burdening national health systems and eliminating the disease will confer public health benefits as well as major cost savings to national health systems. The investments needed, while substantial in the short-term, are time-limited as costs taper off significantly as more countries eliminate the disease. Secondly, there is a strong correlation between the decline in malaria burden and financing. Declining financing for malaria is an imminent threat to malaria elimination, the spread of drug resistance, and regional health security in the Asia-Pacific region. Lastly, malaria is one of the oldest diseases known to mankind: eliminating it will pave the way for purging other ancient infectious diseases of poverty. This investment case provides compelling evidence for the benefits of continued prioritization of funding for malaria, and can be used to develop an advocacy strategy for increased domestic and external funding for the region to reach its goal to be malaria-free by 2030.

## Data availability

### Underlying data

Data used to calibrate and validate the model were sourced from World Malaria Reports (2008–2016) and peer-reviewed literature ^13– 23^. Where there were gaps in the data, these were imputed based on observed changes in reported incidence.

### Extended data

Zenodo: sheetalsilal/METCAP: METCAP Model. https://doi.org/10.5281/zenodo.2575474 ^24^. The following extended data are available:

- Extended Cost Database.docx (Table E1. Cost estimations used in calculations)

Extended data are available under the terms of the Creative Commons Zero "No rights reserved" data waiver (CC0 1.0 Public domain dedication).

## Notes

^1^ The Asia-Pacific region in this report encompasses the 21 malaria-endemic countries as defined by APLMA. Sri Lanka has since been declared as malaria free but still implements prevention of reintroduction activities. Countries include: Afghanistan, Bangladesh, Bhutan, Cambodia, Democratic People’s Republic of Korea (DPRK), India, Indonesia, Lao People’s Democratic Republic (Lao PDR), Malaysia, Myanmar, Nepal, Pakistan, Papua New Guinea (PNG), People’s republic of China, Philippines, Republic of Korea (ROK), Solomon Islands, Sri Lanka, Thailand, Timor Leste, Vanuatu and Vietnam.

^2^ At the 2013 East Asia Summit (EAS), the Asia-Pacific Leaders Malaria Alliance (APLMA) was established to accelerate progress towards a reduction in malaria cases and deaths. In 2014 at the ninth EAS, the APLMA Co- Chairs (the Prime Ministers of Viet Nam and Australia) tabled a recommendation for the Asia-Pacific region to become free of malaria by 2030. EAS Heads of Government agreed to the goal, and tasked APLMA Co- Chairs to present a plan to reach malaria elimination through a “Leaders’ Malaria Elimination Roadmap” ^8^. The APLMA roadmap was presented to Heads of Government during the 10th EAS Meeting in 2015.

^3^ Sri Lanka saw its last indigenous case in 2012 and obtained WHO certification in 2016.

^4^ Projected from estimated cases

^5^ Projected from reported cases
