# Supplementary material for: Malaria elimination transmission and costing in the Asia-Pacific: Developing an investment case
Source: Wellcome Open Res. 2020 Jan 14;4:60. Originally published 2019 Apr 1. [Version 2] doi: 10.12688/wellcomeopenres.14769.2 (PMC6974926; doi:10.12688/wellcomeopenres.14769.2)
Supplement: Peer review history

# Peer review: Reviewer response for version 2

**DOI:** 10.21956/wellcomeopenres.17167.r37577

**Published:** 2020-01-21

**Authors:** Justice Nonvignon

**Affiliations:** 1 Department of Health Policy, Planning and Management, University of Ghana, Accra, Ghana

I have reviewed the revision and author response to my and the other reviewer comments and I find that they have addressed all but one, which I think is critical:

 The title is still confusing and authors chose to maintain that with the excuse that other papers have cited this, which is a weak excuse, in my view. The "Malaria elimination transmission" part of the title is very confusing. Did they mean to say costing of malaria elimination and transmission, what exactly. This is similar to the comment raised by the other reviewer, and I suggest that authors reconsider that. It takes away from the beauty of the paper, I am afraid.

I confirm that I have read this submission and believe that I have an appropriate level of expertise to confirm that it is of an acceptable scientific standard.

# Peer review: Reviewer response for version 1

**DOI:** 10.21956/wellcomeopenres.16095.r36223

**Published:** 2019-09-24

**Authors:** Anuj Tiwari

**Affiliations:** 1 Department of Public Health, Erasmus University Medical Center (Erasmus MC), Rotterdam, The Netherlands

In general, the work is relevant and nicely done. Authors need to bring more clarity on the assumptions and their basis. The modelling studies have many assumptions in general. Kindly improve the sequence of experiments in the methodology section which is now quite unclear on how the model was parameterized with what data. The model inputs should be in the main text in a table with clear referencing and not in the file extension.

 Below are the minor comments in detail. 

1. In the title, “malaria transmission elimination” is confusing because elimination of transmission is a strange combination. Or maybe a comma between transmission and elimination may help.
2. In Cost estimates and model framework “A full description of the model framework is available elsewhere ^9^”. As this paper is very dependent on this model, it is recommended to present the outline/compartments of this model framework in a figure/supplementary file in this paper. 
3. The method section is confusing because authors described the work in the previous and current studies without clear demarcation. It is difficult to understand that what they have done in this study. Mainly, the method section should describe the work done for this paper.
4. “A total of 80 scenarios were simulated, based on 10 different sets of packages of interventions”- in Table 1 only 10 scenarios are mentioned. Kindly change the terminology or describe what these 80 scenarios means in the main text?
5. “Since the model did not distinguish between indigenous and imported cases, we assumed that certain thresholds of cases are imported, which we subtracted from the model outputs”- what is the assumed threshold? Please mention in the text.
6. “From the range of costs generated, we determined the minimum, maximum, median, mean, and other percentiles” - of what? Economic benefit?
7. Results section:- can authors clearly describe the values or assumptions which made business as usual having an increasing trend in the future? As described in the introduction, there is a great success in the past in the reduction of the incidence of malaria in the region with BAU. Based on the historic declining data, I would also expect a decline trend with BAU, but the universal coverage ****will of course be expected to meet the elimination faster.
8. The discussion should be more based on the results whereas now it is discussing out of the scope of this paper, such as where the finances can be arranged in detail.
9. The discussion section does not contain discussion on studies or reports who worked in the same direction or calculated elimination cost in the past.
10. There is no major explanation over the sensitivity analysis results in the discussion section. The graph 6 and 7 needs explanation that total cost is most sensitive to what?

The conclusion section is very weak as it is generic and not based on the results or what has been discussed. If authors can mention two points as conclusion of their results then it will make the paper strong.

I confirm that I have read this submission and believe that I have an appropriate level of expertise to confirm that it is of an acceptable scientific standard.

# Response

**Published:** 2019-10-17

**Authors:** Rima Shretta

We thank the reviewer for his comments and suggestions for improvement.

We have added some more clarity to the assumptions and their basis where this was missing. We have also reviewed the sequencing in the methodology section and made the steps more explicit. We have added the table of model inputs back into the text and will discuss with the journal editors.

With respect to the minor comments:

1. Title: we will change to “The investment case for malaria elimination in the Asia-Pacific”
2. In Cost estimates and model framework: the full description of the transmission model is available in a separate paper the same issue of this journal (they were submitted as a package) so we do not feel that the construct of the model needs to be included here. It has been cross-referenced.
3. This investment case was one part of a larger study in which a transmission model was developed (Silal *et al.*) and an interactive application was developed (Celhay *et al.*). All three papers are included in this series. Nevertheless, we have reviewed the language and made it more explicit what the methodology refers to.
4. Number of scenarios: A total of 80 scenarios were simulated. 10 different sets of intervention packages were simulated with and without resistance; with and without MDA and with and without LLIN scale-up making a total of 80.
5. Threshold of indigenous cases: Using Sri Lanka’s example of achieving elimination status in 2016, but reaching zero indigenous cases in October 2012, the elimination threshold was defined as the incidence per 1000 population at risk that Sri Lanka reported to WHO in 2013, as this is a proxy for the level of imported cases one would expect to see in a country that has reached zero indigenous cases for the first time. This threshold was applied to the population at risk for all 22 countries. This has been described in Silal *et al.* in this issue but details have been added in this paper as well.
6. From the range of costs generated, we determined the minimum, maximum, median, mean, and other percentiles of the economic benefits. This has been added.
7. The BAU scenario is the range of interventions that were being implemented in 2015-the last available data point at the time of analysis. This varies by country as each country was implementing different interventions as outlined in their respective National Strategic Plans. Given the threat of drug resistance already detected in the Asia Pacific, it is not surprising that without increased efforts to combat drug resistance the BAU will no longer favor a downward trend.
8. The scope of this work was to develop an investment case which also includes discussing alternative sources of financing. Nevertheless, we have added more discussion of the results based on the reviewer’s feedback.
9. A discussion on studies or reports who worked in the same direction or calculated elimination cost in the past has been added.
10. A description of the sensitivity analysis has been added.
11. The conclusion has been revised and strengthened.

# Peer review: Reviewer response for version 1

**DOI:** 10.21956/wellcomeopenres.16095.r35700

**Published:** 2019-07-01

**Authors:** Justice Nonvignon, Eric Nsiah-Boateng

**Affiliations:** 1 Department of Health Policy, Planning and Management, University of Ghana, Accra, Ghana; 2 School of Public Health, University of Ghana, Legon, Ghana; 3 National Health Insurance Authority, Accra, Ghana

**Introductory remarks**

 Despite the significant gains made in reducing the burden of malaria in the past two decades, the disease continues to pose a significant public health challenge, especially in sub-Saharan Africa and the Asia-Pacific regions, requiring significant increases in investment, among other efforts, to eliminate the disease once and for all. This “last push” has been elusive due to many factors, some of which the authors clearly explain. Therefore, the manuscript is timely. Nonetheless, the authors are requested to address the following specific comments to improve the article and give readers a fuller understanding of the disease burden in the 22 countries in the region, investment needed to eliminate it and the benefits to reap if malaria is completely eliminated by 2030.

*Title*

 The title of the manuscript could be better phrased; for instance, how about “transmission and cost of malaria elimination in the Asia-Pacific region: developing an investment case"?

*Methods*

 Methods used are largely appropriate. However, the reporting of some of these methods needs further work:

1. The authors are invited to clearly state the design of the study and provide a brief description of the population being studied especially the population at risk (PAR) by country.
2. The authors are also requested to provide a table of disease (malaria) burden and gross national income of each of the 22 countries studied in the region. This comment stems from the statement in the “Discussion” section that suggests that since 2012, the Global Fund has been allocating funds based on these two indicators to the countries in the region towards malaria elimination. This proposed table will give a clearer picture of the disease burden and funds allocated over the years, as well as the deficit/gap to fill. If possible, the amount allocated to each country by the Global fund since 2012 should be added to the table. This proposed table can be placed in the appendix.
3. Following from the above comment and linking it to a statement in the “Discussion” section (page 12, paragraph 3, line 11–13), the authors are kindly requested to tabulate the 22 countries and their World Bank income-defined categories, e.g. LICs, LMICs and UMICs. This can be added to the table suggested in the previous comment.  
4. Although the authors acknowledged the limitation/discrepancies in the reported incidence/prevalence and burden of malaria in the Asia-Pacific region by international, regional and national bodies and other reports/literature, e.g. WHO, IHME, World Malaria Report, they can provide a table of burden of malaria in the region using one source as suggested earlier.
5. Authors make somewhat contradictory statements in the method regarding how costs were estimated. In one instance, they stated that the transmission model was used to estimate costs and benefits while in another instance, they state that they “…built a cost estimation model.” In any case, no description of the models is provided, except that authors invite readers to review other sources; readers should not have to review other sources to get a basic understanding of these models. It is difficult to assess the cost model and inputs. Authors are encouraged to provide brief paragraphs explaining the key components of the cost model used.  
6. It is unclear what informed the identification/separation of activities mentioned under “cost projections” (page 6). For instance, unit cost of vector control may consider supply chain, training. Again, what exactly does “new treatment mean”? Is this just the cost of the new medicine or the use of that medicine to treat cases? And for these, what specific cost items were considered?
7. What was the source of daily productivity of individuals? Again, what was the source of average age of death due to malaria being 40 years for both males and females? Provide references for these.

*Results/discussion*

1. The arguments made by authors relating to graduation of most countries in the region to income levels that will lead to significant reductions in aid (page 12) could be extended, in our view. Authors focus their argument on the (gloomy side of) reductions in development aid while concurring that the economies of these countries could expand, raising domestic financing. While these are true cases, authors could explore the potential of other forms of support e.g. technical support from development agencies that do not necessarily come in the form of aid. For instance, how can “global public goods” help in maintaining momentum for financing towards elimination?
2. The discussions of the manuscript raise important issues worth exploring. However, we believe that those issues could be presented in a more concise form than they are currently presented. For instance, most of the issues revolve around transition in development assistance for health in the region as a result of economic growth, the need for increased domestic financing – not only from government sources but also from private sources – and the opportunities provided by health system efficiencies. We find some of these key issues scattered in the discussion, which could be better organized.  

**Minor comments **

1. As “morbidity” could mean more than just number of cases, authors are encouraged to use “cases averted” in place of “morbidity averted”.
2. The authors can combine paragraph 9 and 10 in page 6 under “Return on investment”.
3. The authors need to correct the following typos and also review the entire document for any inadvertent errors.
  a) Page 5, paragraph 4, line 1: Insert space between “from” and “2018”
  b) Page 5, paragraph 4, line 6: Remove the second “coverage” in the sentence "LLIN coverage of up to 80% coverage”
  c) Page 6, paragraph 4, line 8: Insert space between “the” and “elimination”
  d) Page 9, Paragraph 2, line 5: Insert space between “PAR’ and “in”.
4. The statement “Malaria cases and deaths have declined by more than 50% between 2010 and 2015 in the region’s 22 malaria-endemic countries”, require citation.

*Concluding remarks:*

 The work presented in the manuscript provide important issues that could generate further discussions around mobilizing resources for malaria elimination in the Asia-Pacific region and beyond. Addressing the comments regarding the scientific methods applied could help improve the manuscript.

We confirm that we have read this submission and believe that we have an appropriate level of expertise to confirm that it is of an acceptable scientific standard, however we have significant reservations, as outlined above.

# Response

**Published:** 2019-07-22

**Authors:** Rima Shretta

We thank the reviewers for their detailed comments. Our responses are below.

*Title*

The title of the manuscript could be better phrased; for instance, how about “transmission and cost of malaria elimination in the Asia-Pacific region: developing an investment case"? **We feel that the title should remain as is due to this work having been already cited by the associated papers in this issue as well as other works.**

1.     The authors are invited to clearly state the design of the study and provide a brief description of the population being studied especially the population at risk (PAR) by country.

**We have added the following paragraph at the beginning of the methods section:**

**"The study design incorporated a variety of quantitative methods: numerical and regression techniques to develop a dynamic transmission model to predict the epidemiological impact of various interventions used for malaria control and elimination; and economic analysis to estimate the cost and economic impact of the interventions nationally and regionally. The population studied was the population at risk of malaria estimated by the WHO.  This was computed as the proportion of the population at high and low risk of malaria (provided by the National Malaria Control Programs), applied to the United Nations population estimates to compute the number of people at risk of malaria. A combination of empirical, published data as well as expert opinion were used to develop the investment case".**  

2.     The authors are also requested to provide a table of disease (malaria) burden and gross national income of each of the 22 countries studied in the region. This comment stems from the statement in the “Discussion” section that suggests that since 2012, the Global Fund has been allocating funds based on these two indicators to the countries in the region towards malaria elimination. This proposed table will give a clearer picture of the disease burden and funds allocated over the years, as well as the deficit/gap to fill. If possible, the amount allocated to each country by the Global fund since 2012 should be added to the table. This proposed table can be placed in the appendix.

**The epidemiological data has been added as an excel file in the extended data section. The GDP/GNI per capita already exists in the Extended Data Table 1.**

3.     Following from the above comment and linking it to a statement in the “Discussion” section (page 12, paragraph 3, line 11–13), the authors are kindly requested to tabulate the 22 countries and their World Bank income-defined categories, e.g. LICs, LMICs and UMICs. This can be added to the table suggested in the previous comment.  

**We have added a row in the existing Extended data table 1 to illustrate the World Bank income classification.**

4.     Although the authors acknowledged the limitation/discrepancies in the reported incidence/prevalence and burden of malaria in the Asia-Pacific region by international, regional and national bodies and other reports/literature, e.g. WHO, IHME, World Malaria Report, they can provide a table of burden of malaria in the region using one source as suggested earlier.

**This has been addressed in 2. Above.**

5.     Authors make somewhat contradictory statements in the method regarding how costs were estimated. In one instance, they stated that the transmission model was used to estimate costs and benefits while in another instance, they state that they “…built a cost estimation model.” In any case, no description of the models is provided, except that authors invite readers to review other sources; readers should not have to review other sources to get a basic understanding of these models. It is difficult to assess the cost model and inputs. Authors are encouraged to provide brief paragraphs explaining the key components of the cost model used.

**We have modified the text to reflect this comment. The output of the transmission model was used to estimate costs and benefits.**

6.     It is unclear what informed the identification/separation of activities mentioned under “cost projections” (page 6). For instance, unit cost of vector control may consider supply chain, training. Again, what exactly does “new treatment mean”? Is this just the cost of the new medicine or the use of that medicine to treat cases? And for these, what specific cost items were considered?

**The costs include the cost of delivery. The text has been modified to reflect this.**

7.     What was the source of daily productivity of individuals? Again, what was the source of average age of death due to malaria being 40 years for both males and females? Provide references for these.

**Productivity losses were calculated using the full-income approach which includes the value of the life years and GDP per capita. We used the remaining life years at age 40 from world life expectancy tables. This reference has been added.**

*Results/discussion*

1.     The arguments made by authors relating to graduation of most countries in the region to income levels that will lead to significant reductions in aid (page 12) could be extended, in our view. Authors focus their argument on the (gloomy side of) reductions in development aid while concurring that the economies of these countries could expand, raising domestic financing. While these are true cases, authors could explore the potential of other forms of support e.g. technical support from development agencies that do not necessarily come in the form of aid. For instance, how can “global public goods” help in maintaining momentum for financing towards elimination?

**We have reviewed these sections and added some perspective to the issue of technical assistance and global public good and their contribution to elimination. Nevertheless, these will not fill the gaps in real financing needed to implement interventions relating to commodity procurement or strengthening surveillance systems which tend to be human resource heavy.**

2.     The discussions of the manuscript raise important issues worth exploring. However, we believe that those issues could be presented in a more concise form than they are currently presented. For instance, most of the issues revolve around transition in development assistance for health in the region as a result of economic growth, the need for increased domestic financing – not only from government sources but also from private sources – and the opportunities provided by health system efficiencies. We find some of these key issues scattered in the discussion, which could be better organized.  

**We have tried to reorganize these sections.**

Minor comments

1.     As “morbidity” could mean more than just number of cases, authors are encouraged to use “cases averted” in place of “morbidity averted”. **Noted and this has been changed**.

2.     The authors can combine paragraph 9 and 10 in page 6 under “Return on investment”. **Done**.

3.     The authors need to correct the following typos and also review the entire document for any inadvertent errors. **Done.**

a) Page 5, paragraph 4, line 1: Insert space between “from” and “2018”

b) Page 5, paragraph 4, line 6: Remove the second “coverage” in the sentence "LLIN coverage of up to 80% coverage”

c) Page 6, paragraph 4, line 8: Insert space between “the” and “elimination”

d) Page 9, Paragraph 2, line 5: Insert space between “PAR’ and “in”.

4.     The statement “Malaria cases and deaths have declined by more than 50% between 2010 and 2015 in the region’s 22 malaria-endemic countries”, require citation. **Done**

*Concluding remarks:*

The work presented in the manuscript provide important issues that could generate further discussions around mobilizing resources for malaria elimination in the Asia-Pacific region and beyond. Addressing the comments regarding the scientific methods applied could help improve the manuscript.

**Thank you.**
